# Rapid detection of structural variation in a human genome using nanochannel-based genome mapping technology

**DOI:** 10.1186/2047-217X-3-34

**Published:** 2014-12-30

**Authors:** Hongzhi Cao, Alex R Hastie, Dandan Cao, Ernest T Lam, Yuhui Sun, Haodong Huang, Xiao Liu, Liya Lin, Warren Andrews, Saki Chan, Shujia Huang, Xin Tong, Michael Requa, Thomas Anantharaman, Anders Krogh, Huanming Yang, Han Cao, Xun Xu

**Affiliations:** BGI-Shenzhen, Shenzhen, 518083 China; BioNano Genomics, San Diego, California 92121 USA; Shenzhen Key Laboratory of Transomics Biotechnologies, Shenzhen, 518083 China; Department of Biology, University of Copenhagen, Copenhagen, 2200 Denmark; School of Bioscience and Biotechnology, South China University of Technology, Guangzhou, 511400 China

**Keywords:** Genome mapping, Structural variation, Repeat units, Epstein-Barr virus (EBV) integration

## Abstract

**Background:**

Structural variants (SVs) are less common than single nucleotide polymorphisms and indels in the population, but collectively account for a significant fraction of genetic polymorphism and diseases. Base pair differences arising from SVs are on a much higher order (>100 fold) than point mutations; however, none of the current detection methods are comprehensive, and currently available methodologies are incapable of providing sufficient resolution and unambiguous information across complex regions in the human genome. To address these challenges, we applied a high-throughput, cost-effective genome mapping technology to comprehensively discover genome-wide SVs and characterize complex regions of the YH genome using long single molecules (>150 kb) in a global fashion.

**Results:**

Utilizing nanochannel-based genome mapping technology, we obtained 708 insertions/deletions and 17 inversions larger than 1 kb. Excluding the 59 SVs (54 insertions/deletions, 5 inversions) that overlap with N-base gaps in the reference assembly hg19, 666 non-gap SVs remained, and 396 of them (60%) were verified by paired-end data from whole-genome sequencing-based re-sequencing or *de novo* assembly sequence from fosmid data. Of the remaining 270 SVs, 260 are insertions and 213 overlap known SVs in the Database of Genomic Variants. Overall, 609 out of 666 (90%) variants were supported by experimental orthogonal methods or historical evidence in public databases. At the same time, genome mapping also provides valuable information for complex regions with haplotypes in a straightforward fashion. In addition, with long single-molecule labeling patterns, exogenous viral sequences were mapped on a whole-genome scale, and sample heterogeneity was analyzed at a new level.

**Conclusion:**

Our study highlights genome mapping technology as a comprehensive and cost-effective method for detecting structural variation and studying complex regions in the human genome, as well as deciphering viral integration into the host genome.

**Electronic supplementary material:**

The online version of this article (doi:10.1186/2047-217X-3-34) contains supplementary material, which is available to authorized users.

## Background

A structural variant (SV) is generally defined as a region of DNA 1 kb and larger in size that is different with respect to another DNA sample [1]; examples include inversions, translocations, deletions, duplications and insertions. Deletions and duplications are also referred to as copy number variants (CNVs). SVs have proven to be an important source of human genetic diversity and disease susceptibility [2–6]. Base pair differences arising from SVs occur on a significantly higher order (>100 fold) than point mutations [7, 8], and data from the 1000 Genomes Project show population-specific patterns of SV prevalence [9, 10]. Also, recent studies have firmly established that SVs are associated with a number of human diseases ranging from sporadic syndromes and Mendelian diseases to common complex traits, particularly neurodevelopmental disorders [11–13]. Chromosomal aneuploidies, such as trisomy 21 and monosomy X have long been known to be the cause of Down’s and Turner syndromes, respectively. A microdeletion at 15q11.2q12 has been shown causal for Prader-Willi syndrome [14], and many submicroscopic SV syndromes have been revealed since then [15]. In addition, rare, large *de novo* CNVs were identified to be enriched in autism spectrum disorder (ASD) cases [16], and other SVs were described as contributing factors for other complex traits including cancer, schizophrenia, epilepsy, Parkinson’s disease and immune diseases, such as psoriasis (reviewed in [11] and [12]). With the increasing recognition of the important role of genomic aberrations in disease and the need for improved molecular diagnostics, comprehensive characterization of these genomic SVs is vital for, not only differentiating pathogenic events from benign ones, but also for rapid and full-scale clinical diagnosis.

While a variety of experimental and computational approaches exist for SV detection, each has its distinct biases and limitations. Hybridization-based approaches [17–19] are subject to amplification, cloning and hybridization biases, incomplete coverage, and low dynamic range due to hybridization saturation. Moreover, detection of CNV events by these methods provides no positional context, which is critical to deciphering their functional significance. More recently, high-throughput next generation sequencing (NGS) technologies have been heavily applied to genome analysis based on alignment/mapping [20–22] or *de novo* sequence assembly (SA) [23]. Mapping methods include paired-end mapping (PEM) [20], split-read mapping (SR) [21] and read depth analysis (RD) [22]. These techniques can be powerful, but are tedious and biased towards deletions owing to typical NGS short inserts and short reads [24, 25]. *De novo* assembly methods are more versatile and can detect a larger range of SV types and sizes (0 ~ 25 kb) by pair-wise genome comparison [23–25]. All such NGS-based approaches lack power for comprehensiveness and are heavily biased against repeats and duplications because of short-read mapping ambiguity and assembly collapse [9, 10, 26]. David C. Schwartz’s group promoted optical mapping [27] as an alternative to detect SVs along the genome with restriction mapping profiles of stretched DNA, highlighting the use of long single-molecule DNA maps in genome analysis. However, since the DNA is immobilized on glass surfaces and stretched, the technique suffers from low throughput and non-uniform DNA stretching, resulting in imprecise DNA length measurement and high error rate, hindering its utility and adoption [24, 27–29]. Thus, an effective method to help detect comprehensive SVs and reveal complex genomic regions is needed.

The nanochannel-based genome mapping technology, commercialized as the “Irys” platform, automatically images fluorescently labeled DNA molecules in a massively parallel nanochannel array, and was introduced as an advanced technology [30] compared to other restriction mapping methods because of high-throughput data collection and its robust and highly uniform linearization of DNA in nanochannels. This technology has previously been described and used to map the 4.7-Mb highly variable human major histocompatibility complex (MHC) region [31], as well as for *de novo* assembly of a 2.1-Mb region in the highly complex *Aegilops tauschii* genome [32], lending great promise for use in complete genome sequence analysis. Here, we apply this rapid and high-throughput genome mapping method to discern genome wide SVs, as well as explore complex regions based on the YH (first Asian genome) [33] cell line. The workflow for mapping a human genome on Irys requires no library construction; instead, whole genomic DNA is labeled, stained and directly loaded into nanochannels for imaging. With the current throughput, one can collect enough data for *de novo* assembly of a human genome in less than three days. Additionally, comprehensive SV detection can be accomplished with genome mapping alone, without the addition of orthogonal technologies or multiple library preparations. Utilizing genome mapping, we identified 725 SVs including insertions/deletions, inversions, as well as SVs involved in N-base gap regions that are difficult to assess by current methods. For 50% of these SVs, we detected a signal of variation by re-sequencing and an additional 10% by fosmid sequence-based *de novo* assembly whereas the remainder had no signal by sequencing, hinting at the intractability of detection by sequencing. Detailed analyses showed most of the undetected SVs (80%, 213 out of 270) could be found overlapped in the Database of Genomic Variant (DGV) database indicating their reliability. Genome mapping also provides valuable haplotype information on complex regions, such as MHC, killer cell Immunoglobulin-like receptor (KIR), T cell receptor alpha/beta (TRA/TRB) and immunoglobulin light/heavy locus (IGH/IGL), which can help determine these hyper-variable regions’ sequences and downstream functional analyses. In addition, with long molecule labeling patterns, we were able to accurately map the exogenous virus’ sequence that integrated into the human genome, which is useful for the study of the mechanism of how virus sequence integration leads to serious diseases like cancer.

## Data description

High-molecular weight DNA was extracted from the YH cell line, and high-quality DNA was labeled and run on the Irys system. After excluding DNA molecules smaller than 100 kb for analysis, we obtained 303 Gb of data giving 95× depth for the YH genome (Table 1). For subsequent analyses, only molecules larger than 150 kb (223 Gb, ~70X) were used. *De novo* assembly resulted in a set of consensus maps with an N50 of 1.03 Mb. We performed “stitching” of neighboring genome maps that were fragmented by fragile sites associated with nick sites immediately adjacent to each other. After fragile site stitching, the N50 improved to 2.87 Mb, and the assembly covered 93.0% of the non-N base portion of the human genome reference assembly hg19. Structural variation was classified as a significant discrepancy between the consensus maps and the hg19 *in silico* map. Further analyses were performed for highly repetitive regions, complex regions and Epstein-Barr virus (EBV) integration. Supporting data is available from the *GigaScience* database, GigaDB [34–36].

**Table 1:** **Molecule collection statistics under different length thresholds**

Length cutoff (kb)	No. Molecules	Total length (Gb)	Estimated depth (X)*
100	1,568,969	303	95
120	1,313,832	275	86
150	932,855	223	70
180	659,149	179	56
200	523,146	153	48
300	170,432	68	21
500	22,944	14	4

*Estimated depth based on 3.2 Gb genome size.

## Analyses

### Generation of single-molecule sequence motif maps

Genome maps were generated for the YH cell line by purifying high-molecular weight DNA in a gel plug and labeling at single-strand nicks created by the Nt.BspQI nicking endonuclease. Molecules were then linearized in nanochannel arrays etched in silicon wafers for imaging [31, 32]. From these images, a set of label locations on each DNA molecule defined an individual single-molecule map. Single molecules have, on average, one label every 9 kb and were up to 1 Mb in length. A total of 932,855 molecules larger than 150 kb were collected for a total length of 223 Gb (~70-fold average depth) (Table 1). Molecules can be aligned to a reference to estimate the error rates in the single molecules. Here, we estimated the missing label rate is 10%, and the extra label rate is 17%. Most of the error associated with these reference differences are averaged out in the consensus *de novo* assembly. Distinct genetic features intractable to sequencing technologies, such as long arrays of tandem repeats were observed in the raw single molecules (Additional file 1: Figure S1).

### *De novo*assembly of genome maps from single-molecule data

**Figure 1:**
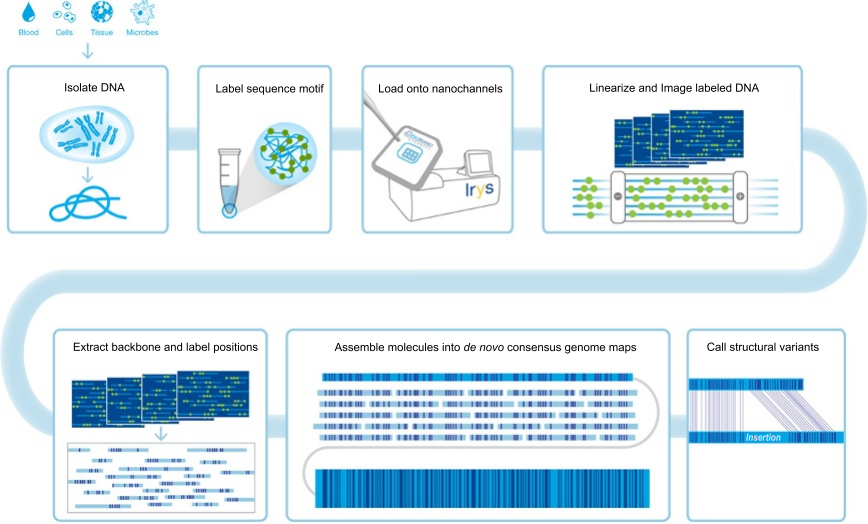
**Flowchart of consensus genome map assembly and structural variant discovery using genome mapping data.**

*S*ingle molecules were assembled *de novo* into consensus genome maps using an implementation of the overlap-layout-consensus paradigm [37]. An overlap graph was constructed by an initial pairwise comparison of all molecules >150 kb, by pattern matching using commercial software from BioNano Genomics. Thresholds for the alignments were based on a p-value appropriate for the genome size (thresholds can be adjusted for different genome sizes and degrees of complexity) to prevent spurious edges. This graph was used to generate a draft consensus map set that was improved by alignment of single molecules and recalculation of the relative label positions. Next, the consensus maps were extended by aligning overhanging molecules to the consensus maps and calculating a consensus in the extended regions. Finally, the consensus maps were compared and merged where patterns matched (Figure 1). The result of this *de novo* assembly is a genome map set entirely independent of known reference or external data. In this case, YH was assembled with an N50 of 1.03 Mb in 3,565 maps and an N50 of 2.87 Mb in 1,634 maps after stitching fragile sites (Additional file 1: Figure S2 and Additional file 1: Table S1). These genome maps define motif positions that occur on every 9 kb on average, and these label site positions have a resolution of 1.45 kb. The standard deviation for interval measurements between two labels varies with length. For example, for a 10 kb interval, the standard deviation (SD) is 502 bp, and for a 100 kb interval, it is 1.2 kb. Consensus genome maps were aligned to an *in silico* Nt.BspQI sequence motif map of hg19. Ninety-nine percent of the genome maps could align to hg19 and they overlap 93% of the non-gap portion of hg19.

### Structural variation analysis

**Figure 2:**
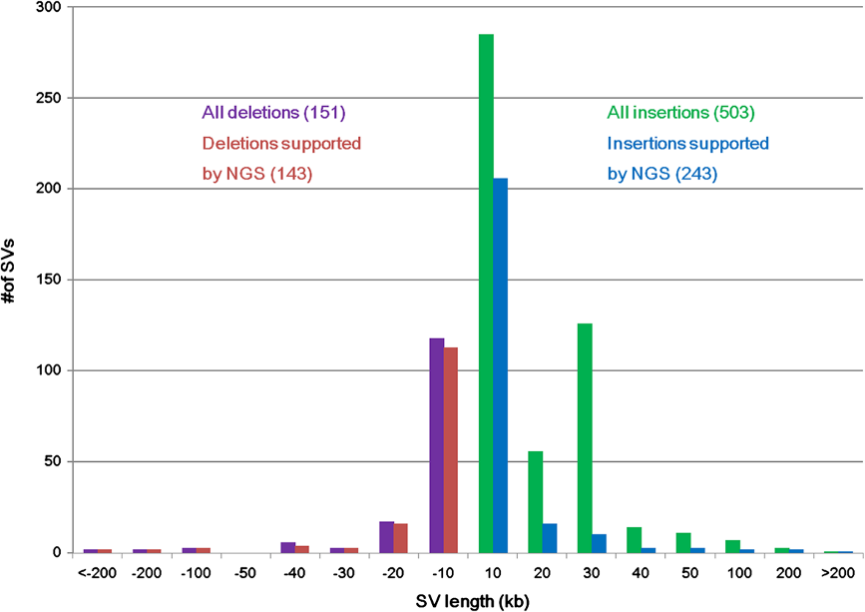
**Size distribution of total detected large insertions (green) and deletions (purple) using genome mapping.** The comparative histogram bars in red and blue respectively represent deletions and insertions supported by NGS. NGS: next-generation sequencing.

Using the genome map assembly as input, we performed structural variation detection (Figure 1), and the genome maps were compared to hg19. Strings of intervals between labels/nick motifs were compared and when they diverged, an outlier p-value was calculated and SVs were called at significant differences (See Methods for details), generating a list of 725 SVs including 59 that overlapped with N-base gaps in hg19 (Additional file 2, Spreadsheet 3). Based on the standard deviation of interval measurements, 1.5 kb is the smallest insertion or deletion that can be confidently measured for an interval of about 10 kb if there is no pattern change. However, if label patterns deviate from the reference, SVs with a net size difference less than 1.5 kb can be detected. Additional file 1: Figure S1 shows three mapping examples (one deletion, one insertion, and one inversion) of gap region SVs. We present these 59 events separately although technically, in those cases, genome mapping detected structural differences between the genome maps and reference regions. For the remaining 666 SVs, 654 of them were insertions/deletions (Figure 2) while 12 were inversions (Additional file 2, Spreadsheet 1 & 2). Out of the 654 insertions/deletions, 503 were defined as insertions and 151 were deletions, demonstrating an enrichment of insertions for this individual with respect to the hg19 reference (Figure 2). Of the 59 SV events that span N-gap regions, 5 of them were inversions. Of the remaining 54 events, 51 were estimated to be shorter than indicated and 3 longer. These gap-region related SVs indicate a specific structure of gap regions of the YH genome compared to the hg19 reference.

In order to validate our SVs, we first cross-referenced them with the public SV database DGV (http://dgv.tcag.ca/dgv/app/home) [38]. For each query SV, we required 50% overlap with records in DGV. We found that the majority of the SVs (583 out of 666; 87.5%) could be found (Additional file 2, Spreadsheet 1 & 2), confirming their reliability. Next, we applied the NGS discordant paired-end mapping and read depth-based methods, as well as fosmid-based *de novo* assembly (See Methods for detail), and as a result, detected an SV signal in 396 (60%, Figure 2) out of 666 SVs by at least one of the two methods (Figure 2, Additional file 2, Spreadsheet 1 & 2). For the remaining 270 SVs, 79% (213 out of 270, Additional file 2, Spreadsheet 1 & 2) were found in the DGV database. Overall, 91% (609 out of 666, Additional file 2, Spreadsheet 1 & 2) of SVs had supporting evidence by retrospectively applied sequencing-based methods or database entries.

We wanted to determine if SVs revealed by genome mapping, but without an NGS supported signal, had unique properties. We firstly investigated the distribution of NGS-supported SVs and NGS-unsupported SVs in repeat-rich and segmental duplication regions. However we did not find significant differences between them (data not shown) which was in concordance with previous findings [27]. We also compared the distribution of insertions and deletions of different SV categories and found that SV events that were not supported by sequencing evidence were 97% (260 out of 268) insertions; in contrast, the SVs that were supported by sequencing evidence were only 61% (243 out of 396, Figure 2, Additional file 2, Spreadsheet 1) insertions showing insertion enrichment (p = 2.2e-16 Chi-squared test, Figure 2) in SVs without sequencing evidence. In addition, we further investigated the novel 57 SVs without either sequencing evidence or database supporting evidence. We found that the genes they covered had important functions, such as ion binding, enzyme activating and so forth, indicating their important role in cellular biochemical activities. Some of the genes like *ELMO1, HECW1, SLC30A8, SLC16A12, JAM3* are reported to be associated with diseases like diabetic nephropathy, lateral sclerosis, diabetes mellitus and cataracts [39], providing valuable foundation for clinical application (Additional file 2, Spreadsheet 1 & 2).

### Highly repetitive regions of the human genome

**Figure 3:**
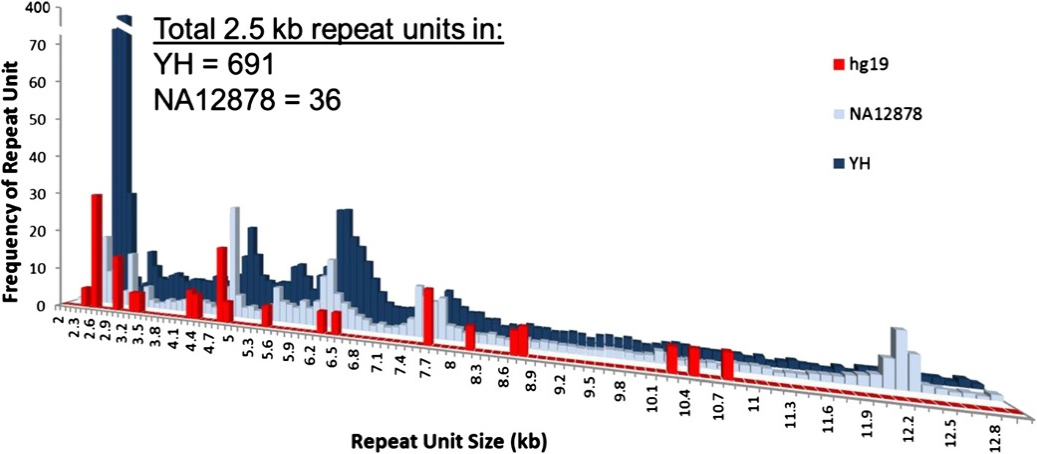
**A plot of repeat units in two human genomes as seen in single molecules.** A repeat unit is defined as five or more equidistant labels. Total units in bins are normalized to the average coverage depth in the genome.

Highly repetitive regions of the human genome are known to be nearly intractable by NGS because short reads are often collapsed, and these regions are often refractory to cloning. We have searched for and analyzed one class of simple tandem repeats (unit size ranging from 2-13 kb) in long molecules derived from the genomes of YH (male) and CEPH-NA12878 (female). The frequencies of these repeating units from both genomes were plotted in comparison with hg19 (Figure 3). We found repeat units across the entire spectrum of sizes in YH and NA12878 while there were only sporadic peaks in hg19, implying an under representation of copy number variation as described in the current reference assembly. Furthermore, we have found a very large peak of approximately 2.5-kb repeats in YH (male, 691 copies) but not in NA19878 (female, 36 copies; Figure 3). This was further supported by additional genome mapping in other males and females demonstrating a consistent and significant quantity of male-specific repeats of 2.5 kb (unpublished). As an example, Additional file 1: Figure S3 shows a raw image of an intact long molecule of 630 kb with two tracts of at least 53 copies and at least 21 copies of 2.5-kb tandem repeats (each 2.5-kb unit has one nick label site, creating the evenly spaced pattern) physically linked by another label-absent putative tandem repeat spanning over 435 kb, and Additional file 1: Figure S4 shows convincing mapping information. Unambiguously elucidating the absolute value and architecture of such complex repeat regions is not possible with other short fragment or hybridization-based methods.

### Complex region analysis using genome mapping

Besides SV detection, genome mapping data also provide abundant information about other complex regions in the genome. For complex regions that are functionally important, an accurate reference map is critical for precise sequence assembly and integration for functional analysis [40–43]. We analyzed the structure of some complex regions of the human genome. They include MHC also called Human leukocyte antigen (HLA), KIR, IGL/IGH, as well as TRA/TRB [44–48]. In the highly variable *HLA-A* and *–C* loci, the YH genome shared one haplotype with the previously typed PGF genome (used in hg19) and also revealed an Asian/YH-specific variant on maps 209 and 153 (Additional file 1: Figure S5), respectively. In the variant haplotype (Map ID 153), there is a large insertion at the *HLA-A* locus while at the HLA-D and RCCX loci, YH had an Asian/YH-specific insertion and a deletion. In addition to the MHC region, we also detected Asian/YH-specific structural differences in KIR (Additional file 1: Figure S6), IGH/IGL (Additional file 1: Figure S7), and TRA/TRB (Additional file 1: Figure S8), compared to the reference genome.

### External sequence integration detection using genome mapping

External viral sequence integration detection is important for the study of diseases such as cancer, but current high-throughput methods are limited in discovering integration break points [49–51]. Although fiber fluorescence in situ hybridization (FISH) was used to discriminate between integration and episomal forms of virus utilizing long dynamic DNA molecules [52], this method was laborious, low-resolution and low-throughput. Thus, long, intact high-resolution single-molecule data provided by genome mapping allows for rapid and effective analysis of which part of the virus sequence has been integrated into the host genome and its localization. We detected EBV integration into the genome of the cell line sample.

**Figure 4:**
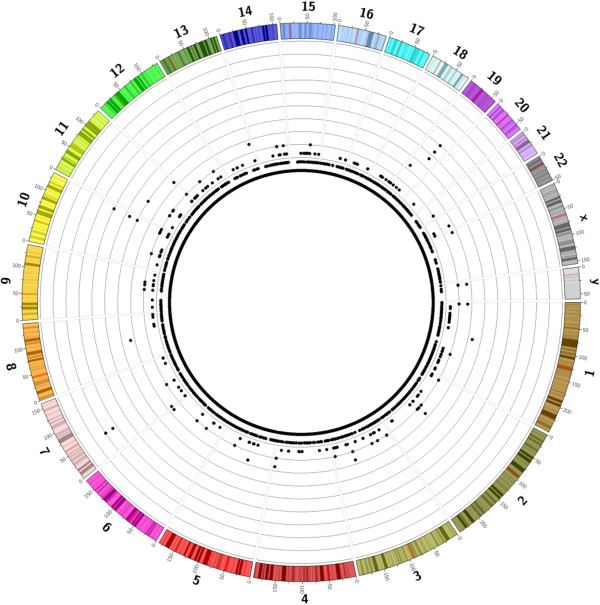
**Circos plot of distribution of integration events throughout YH genome.** The genome was divided into non-overlapping windows of 200 kb. The number of molecules with evidence of integration in each window is plotted with each concentric grey circle representing a two-fold increment in virus detection.

The EBV virus map was assembled *de novo* during the whole genome *de novo* assembly of the YH cell line genome. We mapped the *de novo* EBV map to *in silico* maps from public databases to determine the strain that was represented in the cell line. We found that the YH strain was most closely related, although not identical, to strain B95-8 (GenBank: V01555.2). To detect EBV integration, portions of the aligned molecules extending beyond the EBV map were extracted and aligned with hg19 to determine potential integration sites (Additional file 1: Figure S9). There are 1,340 EBV integration events across the genome (Figure 4). We found that the frequency of EBV integration mapping was significantly lower than the average coverage depth (~70X), implying the DNA sample derived from a clonal cell population is potentially more diverse than previously thought, and that this method could reveal the heterogeneity of a very complex sample population at the single-molecule level. Also, the integrated portion of the EBV genome sequence was detected with a larger fraction towards the tail (Additional file 1: Figure S10). Besides integration events, we also found EBV episome molecules whose single-molecule map could be mapped to the EBV genome, free of flanking human genomic regions.

## Discussion

Structural variants are increasingly frequently shown to play important roles in human health. However, available technologies, such as array-CGH, SNP array and NGS are incapable of cataloguing them in a comprehensive and unbiased manner. Genome mapping, a technology successfully applied to the assembly of complex regions of a plant genome and characterization of structural variation and haplotype differences in the human MHC region, has been adopted to capture the genome-wide structure of a human individual in the current study. Evidence for over 600 SVs in this individual has been provided. Despite the difficulty of SV detection by sequencing methods, the majority of genome map-detected SVs were retrospectively found to have signals consistent with the presence of an SV, validating genome mapping for SV discovery. Approximately 75% of the SVs discovered by genome mapping were insertions; this interesting phenomenon may be a method bias or a genuine representation of the additional content in this genome of Asian descent that is not present in hg19, which was compiled based on genomic materials presumably derived from mostly non-Asians. Analysis of additional genomes is necessary for comparison. Insertion detection is refractory to many existing methodologies [24, 25], so to some extent, genome mapping revealed its distinct potential to address this challenge. Furthermore, functional annotation results of the detected SVs show that 30% of them (Additional file 2, Spreadsheet 1 & 2) affect exonic regions of relevant genes which may cause severe effects on gene function. Gene ontology (GO) analysis demonstrates that these SVs are associated with genes that contribute to important biological processes (Additional file 2, Spreadsheet 1 & 2 and Additional file 1: Figure S11), reflecting that the SVs detected here are likely to affect a large number of genes and may have a significant impact on human health. Genome mapping provides us with an effective way to study the impact of genome-wide SV on human conditions. Some N-base gaps are estimated to have longer or shorter length or more complex structurally compared to hg19, demonstrating that genome mapping is useful for improving the human and other large genome assemblies. We also present a genome-wide analysis of short tandem repeats in individual human genomes and structural information and differences for some of the most complex regions in the YH genome. Independent computational analysis has been performed to discern exogenous viral insertions, as well as exogenous episomes. All of these provide invaluable insights into the capacity of genome mapping as a promising new strategy for research and clinical application.

The basis for the genome mapping technology that enables us to effectively address shortcomings of existing methodologies is the use of motif maps derived from extremely long DNA molecules hundreds of kb in length. Using these motif maps, we are able to also access challenging loci where existing technologies fail. Firstly, global structural variations were easily and quickly detected. Secondly, evidence for a deletion bias which is commonly observed with both arrays and NGS technology, is absent in genome mapping. In fact, we observe more insertions than deletions in this study. Thirdly, for the first time, we are able to measure the length of regions of the YH genome that represent gaps in the human reference assembly. Fourthly, consensus maps could be assembled in highly variable regions in the YH genome which are important for subsequent functional analysis. Finally, both integrated and un-integrated EBV molecules are identified, and potential sub-strains differentiated, and the EBV genome sequence that integrated into the host genome was obtained directly. This information was previously inaccessible without additional PCR steps or NGS approaches [50]. All in all, we demonstrated advantages and strong potential of the genome mapping technology based on nanochannel arrays to help overcome problems that have severely limited our understanding of the human genome.

In addition to the advantages this study reveals about the genome mapping technology, aspects that need to be improved also are highlighted. As genome mapping technology generates sequence-specific motif-labeled DNA molecules and analyzes these motif maps using an overlap-layout-consensus algorithm, subsequent performance and resolution largely depends on motif density (any individual event endpoints can only be resolved to the nearest restriction sites). For example, the EBV integration analysis in this study was more powerful in the high-density regions (Additional file 1: Figure S10). Hence, higher density labeling methods to increase the information density that may promote even higher accuracy and unbiased analysis of genomes are currently being further developed. When data from genome mapping is combined with another source of information, one can achieve even higher resolution for each event. In addition, reducing random errors like extra restriction sites, missing restriction sites and size measurement is important for subsequent analysis. Finally, improvements to the SV detection algorithm will provide further discovery potential, and balanced reciprocal translocations can be identified in genome maps generated from cancer model genomes (personal communication, Michael Rossi).

The throughput and speed of a technology remains one of the most important factors for routine use in clinical screening as well as scientific research. At the time of manuscript submission, genome mapping of a human individual could be accomplished with fewer than three nanochannel array chips in a few days. It is anticipated that a single nanochannel chip would cover a human size genome in less than one day within 6 months, facilitating new studies aimed at unlocking the inaccessible portions of the genome. In this way, genome mapping has an advantage over the use of multiple orthogonal methods that are often used to detect global SVs. Thus, it is now feasible to conduct large population-based comprehensive SV studies efficiently on a single platform.

## Methods

### High-molecular weight DNA extraction

High-molecular weight (HMW) DNA extraction was performed as recommended for the CHEF Mammalian Genomic DNA Plug Kit (BioRad #170-3591). Briefly, cells from the YH or NA12878 cell lines were washed with 2x with PBS and resuspended in cell resuspension buffer, after which 7.5 × 10^5^ cells were embedded in each gel plug. Plugs were incubated with lysis buffer and proteinase K for four hours at 50°C. The plugs were washed and then solubilized with GELase (Epicentre). The purified DNA was subjected to four hours of drop dialysis (Millipore, #VCWP04700) and quantified using Nanodrop 1000 (Thermal Fisher Scientific) and/or the Quant-iT dsDNA Assay Kit (Invitrogen/Molecular Probes).

### DNA labeling

DNA was labeled according to commercial protocols using the IrysPrep Reagent Kit (BioNano Genomics, Inc). Specifically, 300 ng of purified genomic DNA was nicked with 7 U nicking endonuclease Nt.BspQI (New England BioLabs, NEB) at 37°C for two hours in NEB Buffer 3. The nicked DNA was labeled with a fluorescent-dUTP nucleotide analog using Taq polymerase (NEB) for one hour at 72°C. After labeling, the nicks were ligated with Taq ligase (NEB) in the presence of dNTPs. The backbone of fluorescently labeled DNA was stained with YOYO-1 (Invitrogen).

### Data collection

The DNA was loaded onto the nanochannel array of BioNano Genomics IrysChip by electrophoresis of DNA. Linearized DNA molecules were then imaged automatically followed by repeated cycles of DNA loading using the BioNano Genomics Irys system.

The DNA molecules backbones (YOYO-1 stained) and locations of fluorescent labels along each molecule were detected using the in-house software package, IrysView. The set of label locations of each DNA molecule defines an individual single-molecule map.

### *De novo*genome map assembly

Single-molecule maps were assembled *de novo* into consensus maps using software tools developed at BioNano Genomics. Briefly, the assembler is a custom implementation of the overlap-layout-consensus paradigm with a maximum likelihood model. An overlap graph was generated based on pairwise comparison of all molecules as input. Redundant and spurious edges were removed. The assembler outputs the longest path in the graph and consensus maps were derived. Consensus maps are further refined by mapping single-molecule maps to the consensus maps and label positions are recalculated. Refined consensus maps are extended by mapping single molecules to the ends of the consensus and calculating label positions beyond the initial maps. After merging of overlapping maps, a final set of consensus maps was generated and used for subsequent analysis. Furthermore, we applied a “stitching” procedure to join neighboring genome maps. Two adjacent genome maps would be joined together if the junction a) was within 50 kb apart, b) contained at most 5 labels, c) contained, or was within 50 kb from, a fragile site, and d) also contained no more than 5 unaligned end labels. If these criteria were satisfied, the two genome maps would be joined together with the intervening label patterns taken from the reference *in silico* map.

### Structural variation detection

Alignments between consensus genome maps and the hg19 *in silico* sequence motif map were obtained using a dynamic programming approach where the scoring function was the likelihood of a pair of intervals being similar [53]. Likelihood is calculated based on a noise model which takes into account fixed sizing error, sizing error which scales linearly with the interval size, mis-aligned sites (false positives and false negatives), and optical resolution. Within an alignment, an interval or range of intervals whose cumulative likelihood for matching the reference map is worse than 0.01 percent chance is classified as an outlier region. If such a region occurs between highly scoring regions (p-value of 10e^-6^), an insertion or deletion call is made in the outlier region, depending on the relative size of the region on the query and reference maps. Inversions are defined if adjacent match-groups between the genome map and reference are in reverse relative orientation.

### Signals refined by re-sequencing and *de novo*assembly based methods

In order to demonstrate the capacity of genome mapping for the detection of large SVs, we tested the candidate SVs using whole-genome paired-end 100 bp sequencing (WGS) data with insert sizes of 500 bp and fosmid sequence based *de novo* assembly result. SVs were tested based on the expectation that authentic SVs would be supported by abnormally mapped read pairs, and that deletions with respect to the reference should have lower mapped read depth than average [20, 22, 23]. We performed single-end/(paired-end + single-end) reads ratio (sp ratio) calculations at the whole-genome level to assign an appropriate threshold for abnormal regions as well as depth coverage. We set sp ratio and depth cutoff thresholds based on the whole genome data to define SV signals. Insertions with aberrant sp ratio and deletions with either sp ratio or abnormal depth were defined to be a supported candidate.

We also utilized fosmid-based *de novo* assembly data to search for signals supporting candidate SVs. We used contigs and scaffolds assembled from short reads to check for linearity between a given assembly and hg19 using LASTZ [54]. WGS-based and fosmid-based SV validation showed inconsistency and/or lack of saturation as each supported unique variants (Additional file 1: Figure S2) [24].

### EBV integration detection

Single-molecule maps were aligned with a map generated *in silico* based on the EBV reference sequence (strain B95-8; GenBank: V01555.2). Portions of the aligned molecules extending beyond the EBV map were extracted and aligned with hg19 to determine potential integration sites.

### Availability of supporting data

The data sets supporting the results of this article is available in the *GigaScience* GigaDB, repository [55]. See the individual GigaDB entries for the YH Bionano data [35] and YH fosmid validation data [36], which is also available in the SRA [PRJEB7886].

## Electronic supplementary material

Additional file 1: Figure S1: Comparison of consensus genome maps and hg19 reference across gap regions. **Figure S2.** Consensus genome map coverage of human reference assembly (hg19). **Figure S3.** Examples of repetitive sequence detected in intact single molecules by genome mapping. **Figure S4.** Consensus genome map compared to hg19 in a long tandem repeat region. **Figure S5.** Consensus genome maps compared to hg19 in the MHC region. **Figure S6.** Consensus genome maps compared to hg19 in the KIR region. **Figure S7.** Consensus genome maps compared to hg19 in the IGH and IGL regions. **Figure S8.** Consensus genome maps compared to hg19 in the TRA and TRB regions. **Figure S9.** Single-molecule alignment to EBV *in silico* motif map (strain B95-8) showing evidence of strain variation and heterogeneous integration. **Figure S10.** Distribution of integrated portions of the EBV genome. **Figure S11.** GO annotations of genes within called SVs. **Table S1.** Summary of consensus genome map assembly. (DOCX 4 MB)

Additional file 2: **Spreadsheet 1.** Summary of insertions and deletions detected in BioNano genome maps. **Spreadsheet 2:** Summary of inversions detected in BioNano genome maps. **Spreadsheet 3:** Validation of insertions and deletions detected in BioNano genome maps. (XLSX 125 KB)

## Abbreviations

Array-CGH: Array-based comparative genomic hybridization
AS: De novo sequence assembly
ASD: Autism spectrum disorder
BCR: B cell receptor
CNV: Copy number variant
DGV: Database of genomic variants
EBV: Epstein-Barr virus
FISH: Fluorescence in situ hybridization
GO: Gene ontology
HLA: Human leukocyte antigen
HMW: High-molecular weight
IGH: Immunoglobulin heavy locus
IGL: Immunoglobulin light locus
KIR: Killer cell immunoglobulin-like receptor
LRC: Leukocyte Receptor Complex
MHC: Major histocompatibility complex
NGS: Next-generation sequencing
PCR: Polymerase chain reaction
PEM: Pair-end mapping
RD: Read depth
SNP: Single nucleotide polymorphism
SR: Split read
SV: Structural variation
TCR: T cell receptor
TRA: T cell receptor alpha locus
TRB: T cell receptor beta locus
WGS: Whole-genome sequencing
YH: YanHuang.
