# Supplementary material for: Rapid detection of structural variation in a human genome using nanochannel-based genome mapping technology
Source: Gigascience. 2014 Dec 30;3:34. doi: 10.1186/2047-217X-3-34 (PMC4322599; doi:10.1186/2047-217X-3-34)
Supplement: Supplementary file 1 — Additional file 1: Figure S1: Comparison of consensus genome maps and hg19 reference across gap regions. Figure S2. Consensus genome map coverage of human reference assembly (hg19). Figure S3. Examples of repetitive sequence detected in intact single molecules by genome mapping. Figure S4. Consensus genome map compared to hg19 in a long tandem repeat region. Figure S5. Consensus genome maps compared to hg19 in the MHC region. Figure S6. Consensus genome maps compared to hg19 in the KIR region. Figure S7. Consensus genome maps compared to hg19 in the IGH and IGL regions. Figure S8. Consensus genome maps compared to hg19 in the TRA and TRB regions. Figure S9. Single-molecule alignment to EBV in silico motif map (strain B95-8) showing evidence of strain variation and heterogeneous integration. Figure S10. Distribution of integrated portions of the EBV genome. Figure S11. GO annotations of genes within called SVs. Table S1. Summary of consensus genome map assembly. (DOCX 4 MB) [file 13742_2014_59_MOESM1_ESM.docx]

Rapid detection of structural variation in a Human genome using nanochannel-based genome mapping technology

Hongzhi Cao^1,3,4,6^, Alex R. Hastie^2,6^, Dandan Cao^1,3,6^, Ernest T. Lam^2,6^ , Yuhui Sun^1,5^, Haodong Huang^1,5^, Xiao Liu^1^, Liya Lin^1,5^, Warren Andrews^2^, Saki Chan^2^, Shujia Huang^1^, Xin Tong^1^, Michael Requa^2^, Thomas Anantharaman^2^, Anders Krogh^4^, Huanming Yang^1,3^, Han Cao^2 *^, Xun Xu^1,3 *^

^1^BGI-Shenzhen, Shenzhen, 518083, China

^2^BioNano Genomics, San Diego, California, 92121, United States of America

^3^Shenzhen Key Laboratory of Transomics Biotechnologies, Shenzhen, 518083, China

^4^Department of Biology, University of Copenhagen, Copenhagen, 2200, Denmark

^5^School of Bioscience and Biotechnology, South China University of Technology, Guangzhou, 511400, China

^6^These authors contributed equally to this work.

*Correspondence should be addressed to X.X. ([xuxun@genomics.cn](mailto:xuxun@genomics.cn)) and H.C. ([han@bionanogenomics.com](mailto:han@bionanogenomics.com))

**Supplementary Figure and Table**

**Figures:**

**Supp Figure 1:** Comparison of consensus genome maps and hg19 reference across gap regions. Sizing of and assembly around the gap regions are inaccurate; differences between the genome maps and the reference appear as SV calls. The green bars represent the hg19 *in silico* motif map; the blue bars represent consensus genome maps. The vertical black bands are nick motifs/labels, and the lines connecting the blue and green bars indicate matches between labels. Examples of a deletion, insertion, and inversion are shown here.

Deletion [chr1:3835343-4014590]; gap region [chr1:3845269-3995268]

**
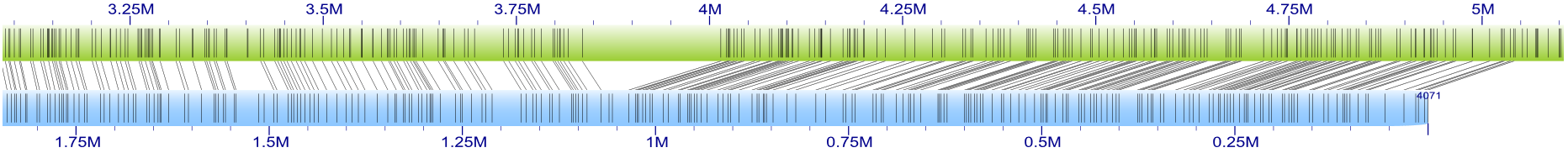
**

Insertion [chr6:95,669,899-95,832,644]; gap region [chr6:95,680,544-95,830,543]

**
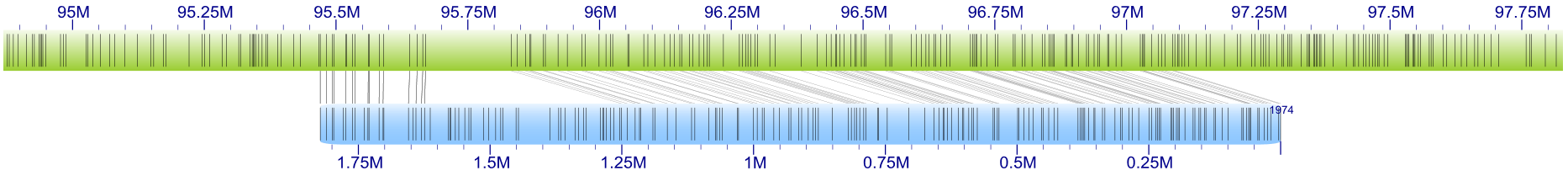
**

Inversion [chr7:142,043,546-142,099,092]; gap region [chr7:142,048,196-142,098,195]

**
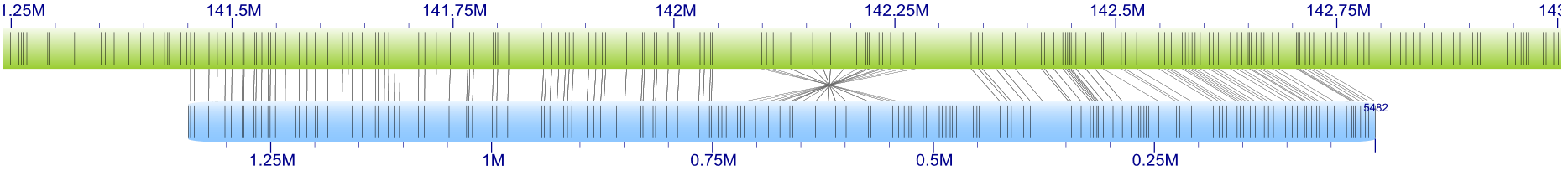
**

**Supp Figure 2:** Consensus genome map coverage of human reference assembly (hg19). The ideogram shows the overlap of the hg19 reference with consensus genome maps in blue. N-base gaps are shown in grey.


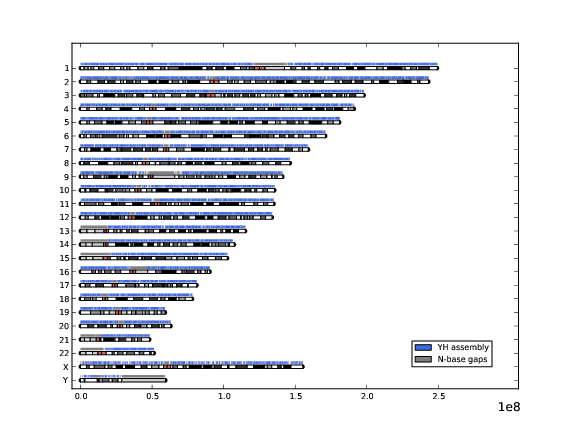


**Supp Figure 3:** Examples of repetitive sequence detected in intact single molecules by genome mapping. A single DNA molecule is shown with labels at 2.5 kb intervals, representing a long tandem repeat structure. Two arrays of 2.5 kb repeats are separated by 435 kb of unlabeled sequence. This 2.5 kb repeat was found to very abundant in the human genome.

**Supp Figure 4:** Consensus genome map compared to hg19 in a long tandem repeat region. The green bars represent the hg19 *in silico* motif map; the blue bar represents the consensus genome map. There is strong molecule support for the long tandem repeat.

**
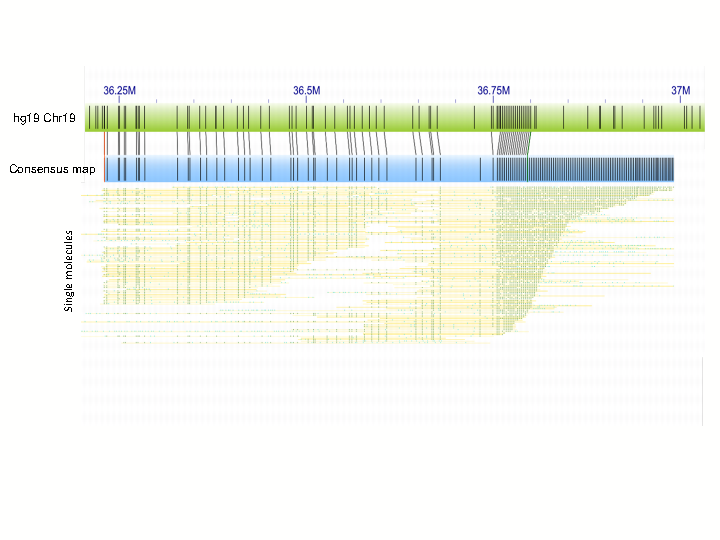
**

**Supp Figure 5:** Consensus genome maps compared to hg19 in the MHC region. The green bars represent the hg19 *in silico* motif map; the blue bars represent consensus genome maps. Large SVs can be seen in the RCCX, HLA-D and *HLA-A* regions. The Cox and PGF genome maps are shown below for the *HLA-A* region. HLA: human leukocyte antigen; RCCX: RP-C4-CYP21-TNX module.


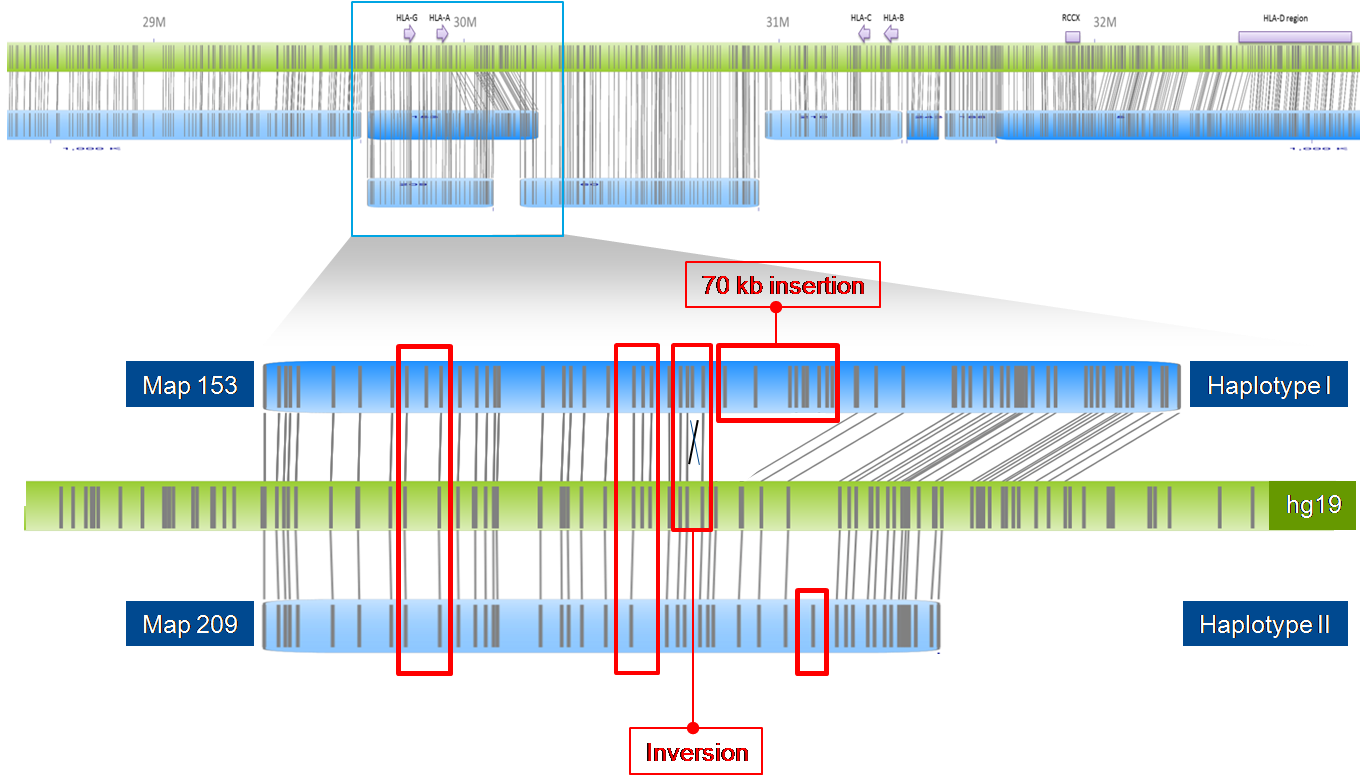


**Supp Figure 6:** Consensus genome maps compared to hg19 in the KIR region. The green bars represent the hg19 *in silico* motif map; the blue bars represent consensus genome maps. The YH genome map shows a huge variation relative to hg19 and HuRef human reference sequences. KIR: killer cell immunoglobulin-like receptor.


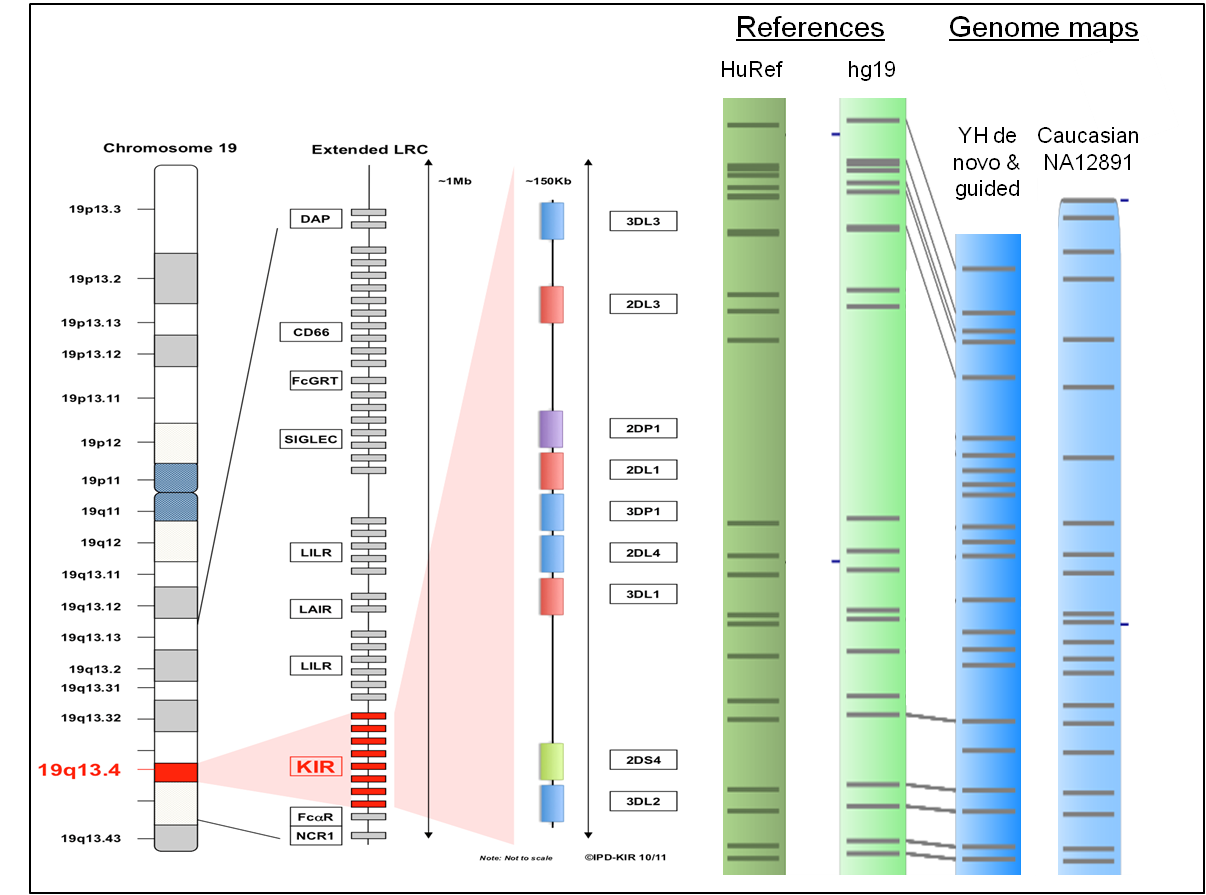


**Supp Figure 7:** Consensus genome maps compared to hg19 in the IGH and IGL regions. The green bars represent the hg19 *in silico* motif map; the blue bars represent consensus genome maps. IGH: immunoglobulin heavy locus ; IGL: immunoglobulin light locus

a


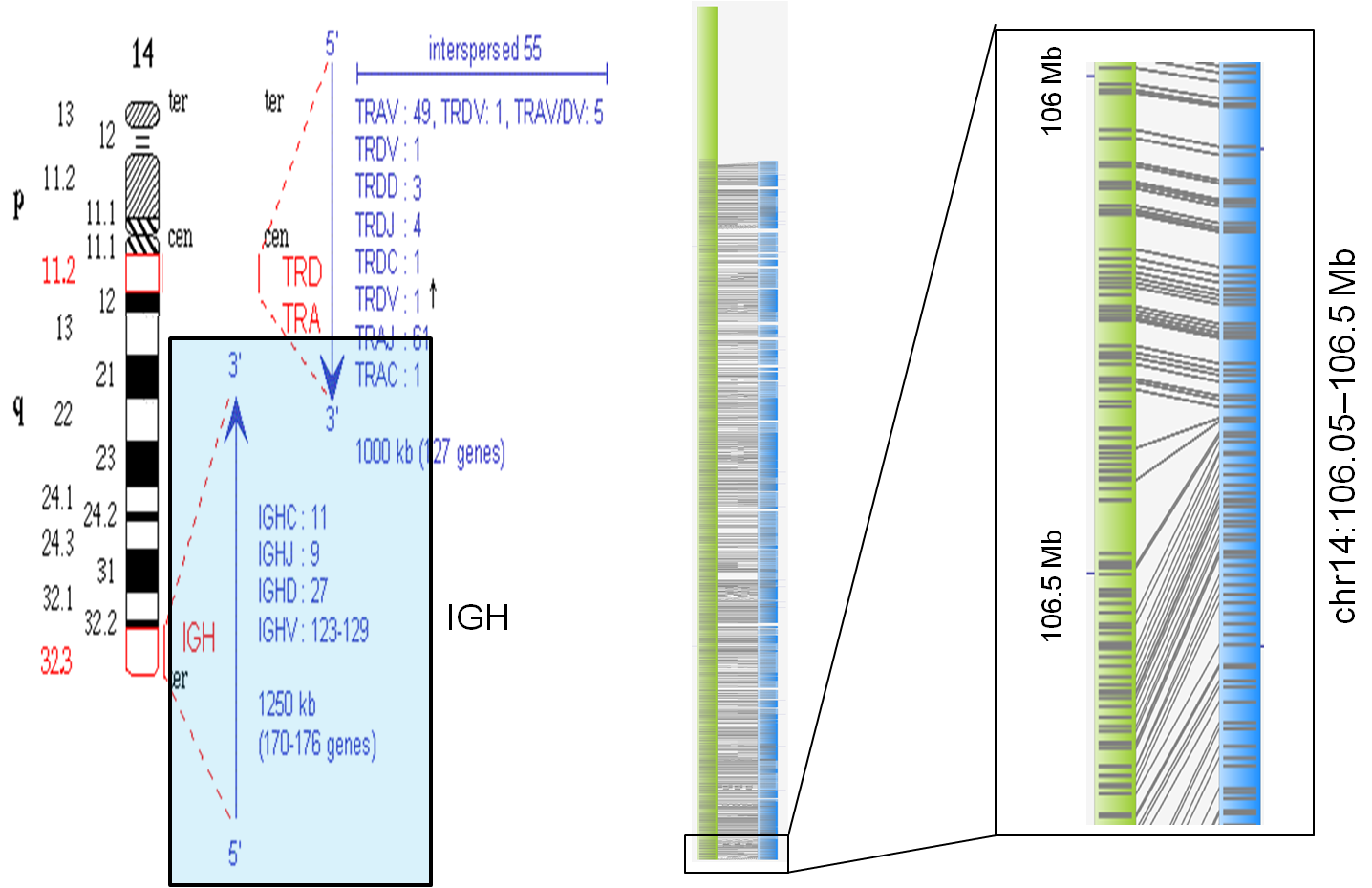


b


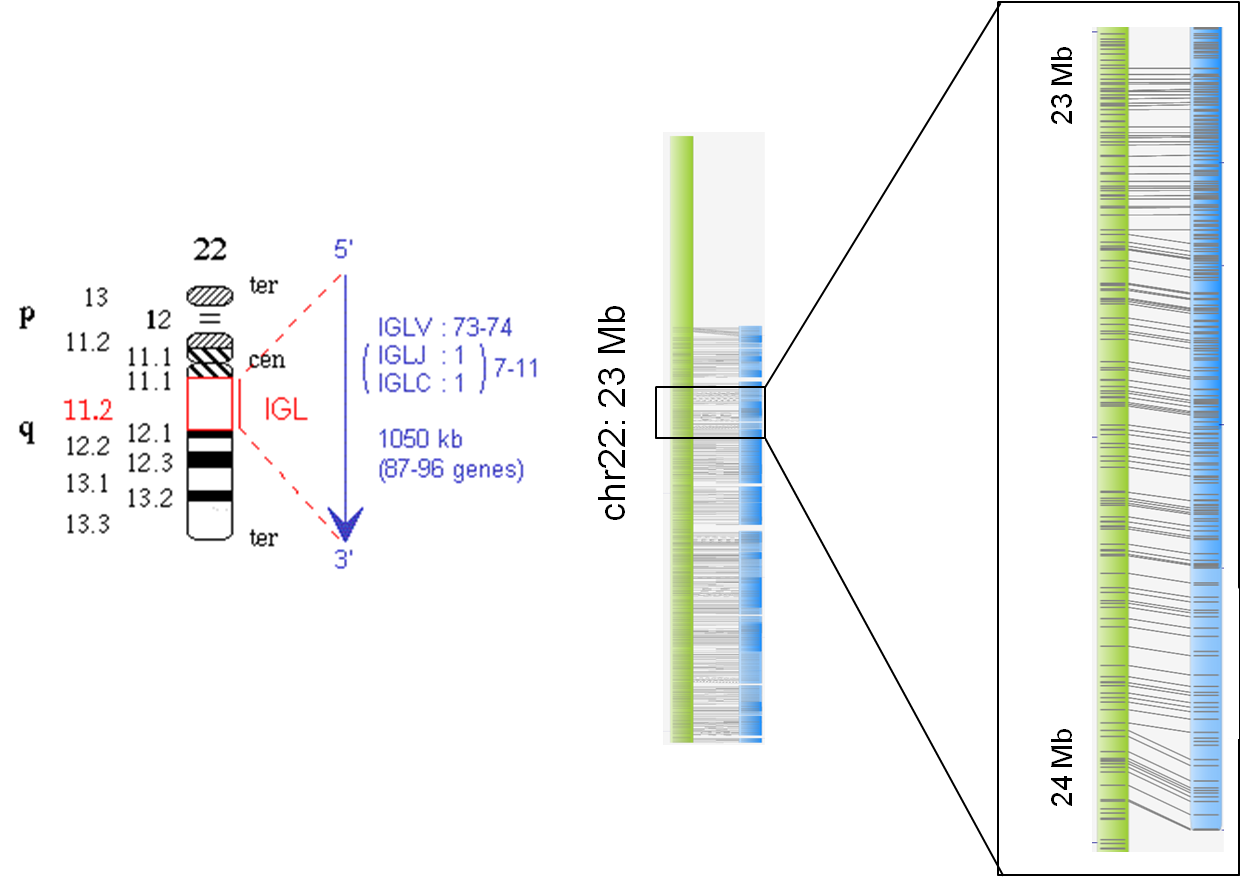


**Supp Figure 8:** Consensus genome maps compared to hg19 in the TRA and TRB regions. The green bars represent the hg19 *in silico* motif map; the blue bars represent consensus genome maps. TRA: T cell receptor alpha locus; TRB: T cell receptor beta locus.

a


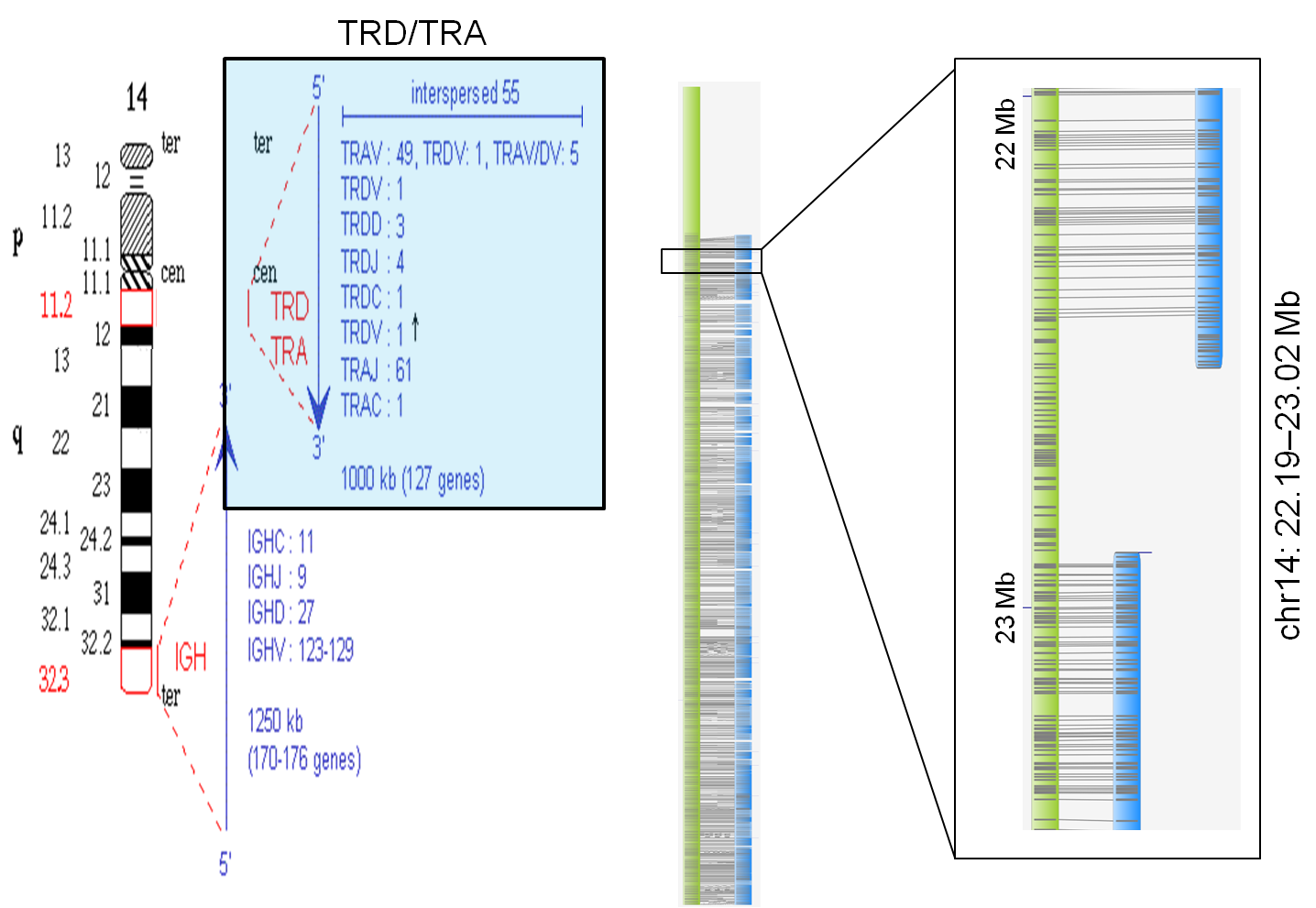


b


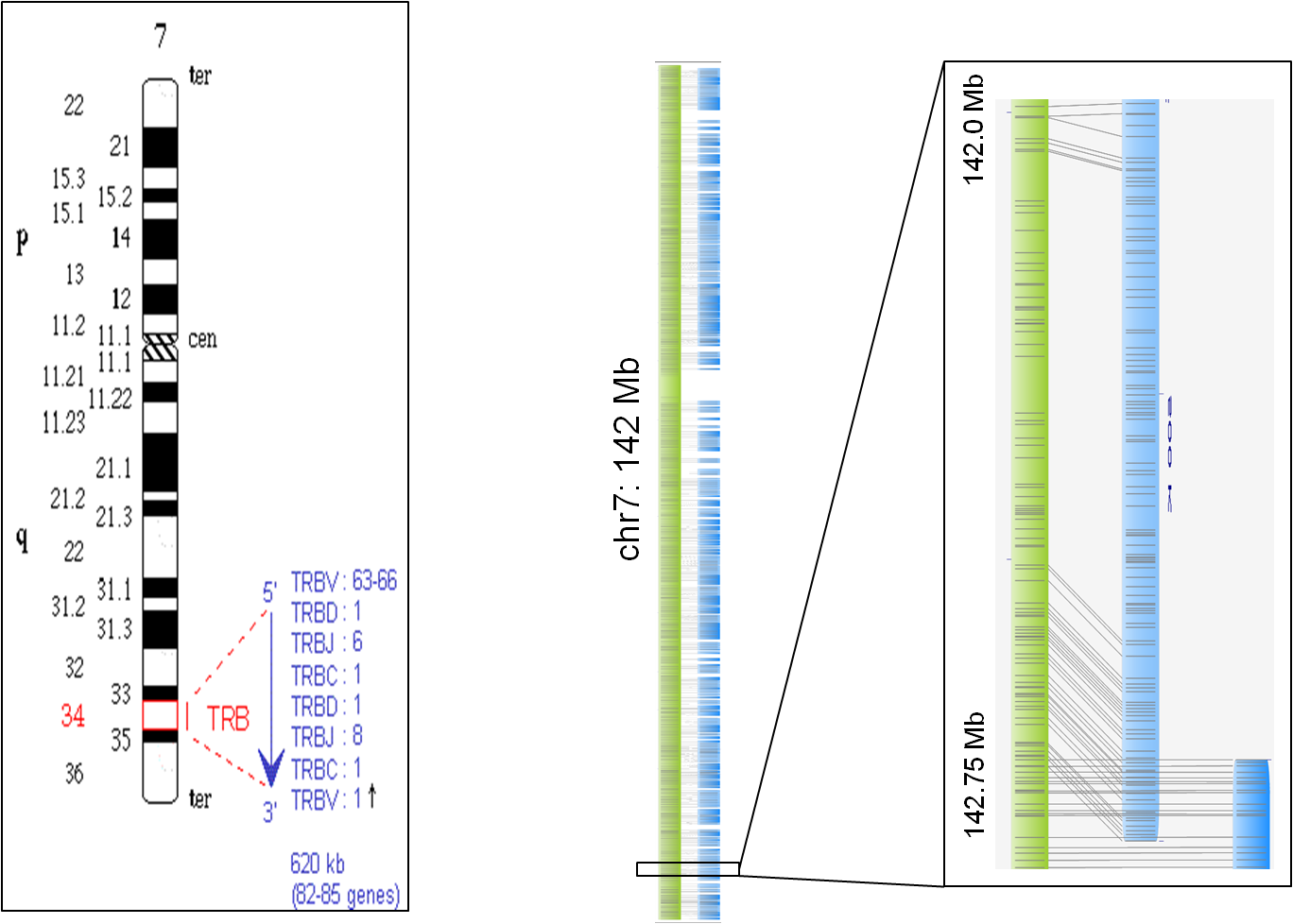


**Supp Figure 9:** Single-molecule alignment to EBV *in silico* motif map (strain B95-8) showing evidence of strain variation and heterogeneous integration. Single molecules (yellow bars with green labels) were aligned with the EBV map (blue bar). Two copies of the EBV map were used as reference to account for the circular nature of the EBV genome. The flanking sequence that extends beyond the EBV map shows no clear consensus, suggesting that there is significant heterogeneity in the cell population. EBV: Epstein-Barr virus.


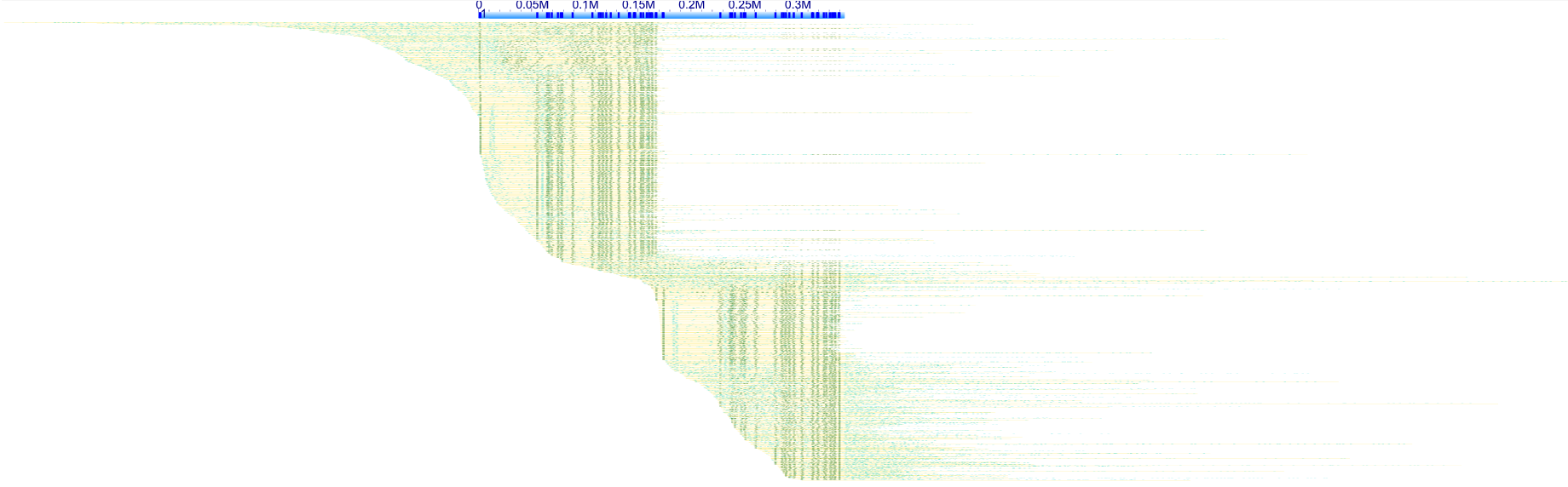


**Supp Figure 10:** Distribution of integrated portions of the EBV genome. EBV: Epstein-Barr virus
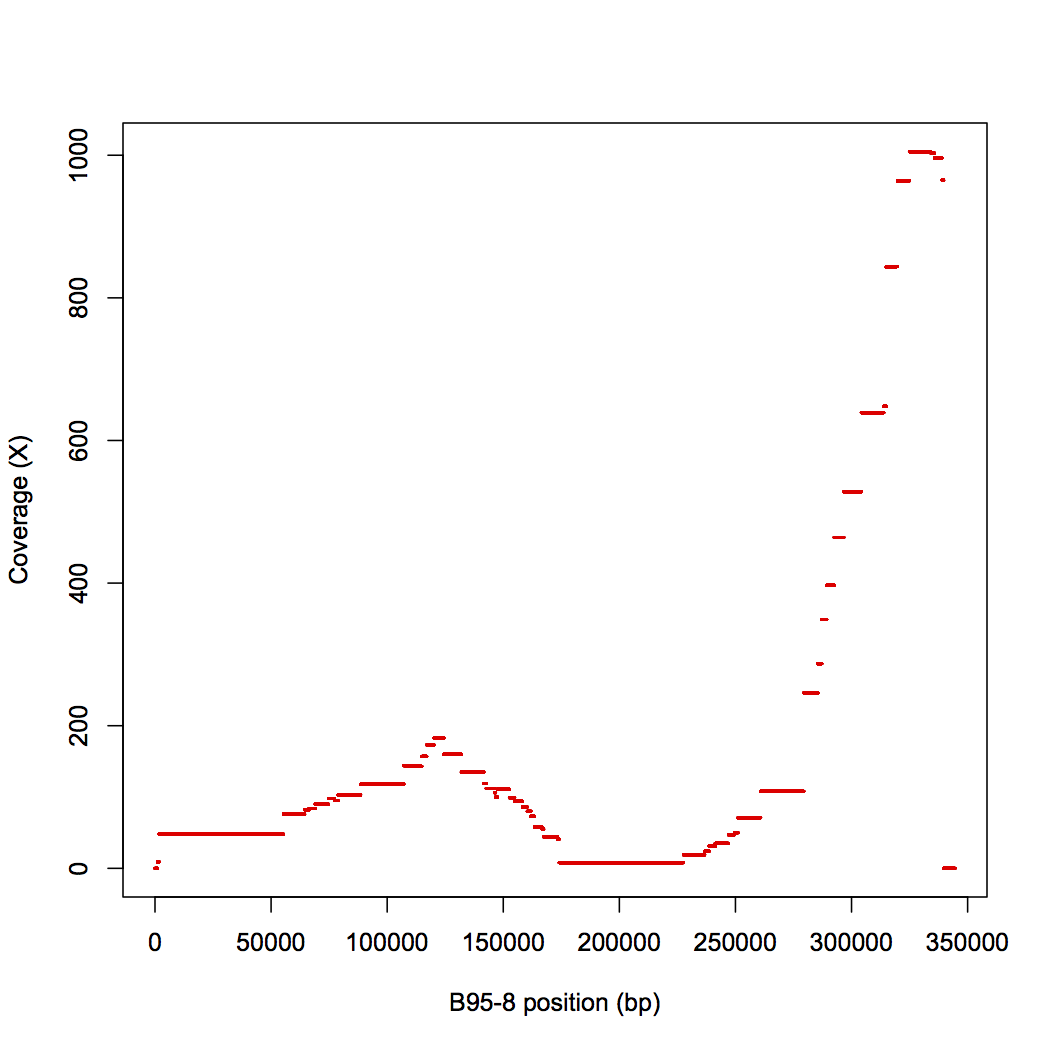


**Supp Figure 11:** GO annotations of genes within called SVs. GO: gene ontology.
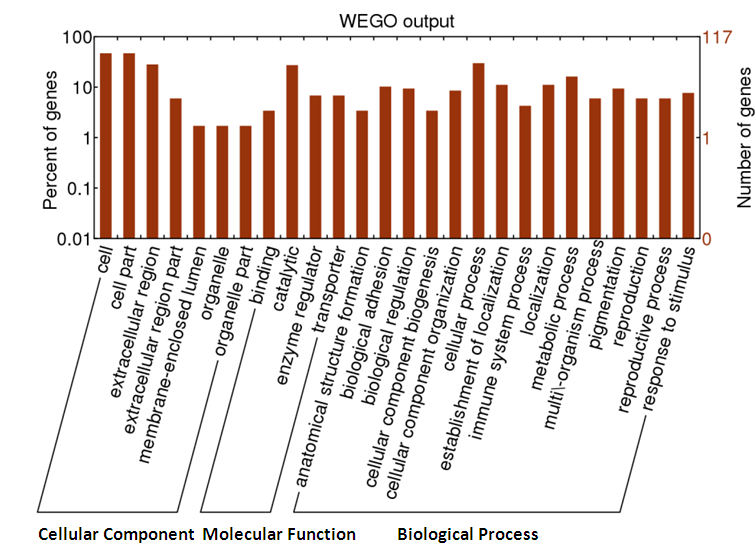


**Tables**

**Supp Table 1:** Summary of consensus genome map assembly

|  | **Pre-stitch** | **Post-stitch** |
| --- | --- | --- |
| **Number of maps** | 3,565 | 1,634 |
| **Min length (bp)** | 90,350 | 90,350 |
| **Median length (bp)** | 599,630 | 1,096,601 |
| **Mean length (bp)** | 781,695 | 1,712,980 |
| **N50 length (bp)** | 1,027,446 | 2,868,628 |
| **Max length (bp)** | 4,956,529 | 11,771,806 |
| **Total length (bp)** | 2,786,743,736 | 2,799,008,620 |
